# *Mycobacterium tuberculosis* Mce3R TetR-like Repressor Forms an Asymmetric Four-Helix Bundle and Binds a Nonpalindrome Sequence†

**DOI:** 10.1021/acschembio.4c00687

**Published:** 2024-11-15

**Authors:** Navanjalee T. Panagoda, Gábor Balázsi, Nicole S. Sampson

**Affiliations:** ‡ Department of Chemistry, Stony Brook University, Stony Brook, New York 11794-3400, United States; § The Louis and Beatrice Laufer Center for Physical and Quantitative Biology, Stony Brook University, Stony Brook, New York 11794-5252, United States; ∥ Department of Biomedical Engineering, Stony Brook University, Stony Brook, New York 11794-2581, United States; ⊥ Department of Chemistry, University of Rochester, Rochester, New York 14627-0216, United States

## Abstract

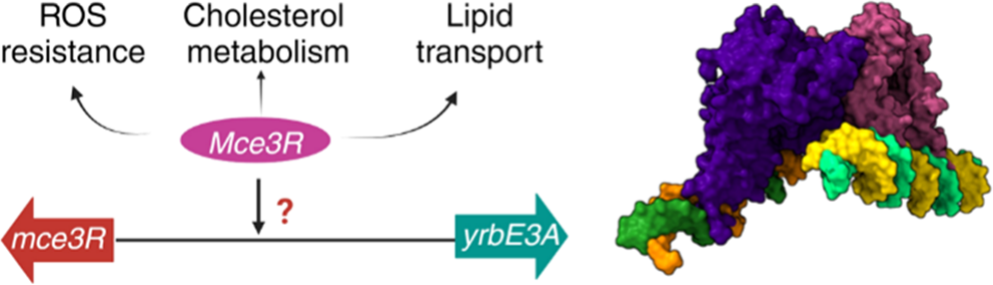

*Mycobacterium tuberculosis* (*Mtb*), the causative agent of tuberculosis, is a major global health concern. TetR family repressors (TFRs) are important for *Mtb*’s adaptation to the human host environment. Our study focuses on one notable *Mtb* repressor, Mce3R, composed of an unusual double TFR motif. Mce3R-regulated genes encode enzymes implicated in cholesterol metabolism, resistance against reactive oxygen species, and lipid transport activities important for *Mtb* survival and persistence in the host and for the cellular activity of a 6-azasteroid derivative. Here, we present the structure of Mce3R bound to its DNA operator, unveiling a unique asymmetric assembly previously unreported. We obtained a candidate DNA-binding motif through MEME motif analysis, comparing intergenic regions of *mce3R* orthologues and identifying nonpalindromic regions conserved between orthologues. Using an electrophoretic mobility shift assay (EMSA), we confirmed that Mce3R binds to a 123-bp sequence that includes the predicted motif. Using scrambled DNA and DNA oligonucleotides of varying lengths with sequences from the upstream region of the *yrbE3A* (*mce3*) operon, we elucidated the operator region to be composed of two Mce3R binding sites, each a 25-bp asymmetric sequence separated by 53 bp. Mce3R binds with a higher affinity to the downstream site with a *K*_d_ of 2.4 ± 0.7 nM. The cryo-EM structure of Mce3R bound to the 123-bp sequence was refined to a resolution of 2.51 Å. Each Mce3R monomer comprises 21 α-helices (α1-α21) folded into an asymmetric TFR-like structure with a core asymmetric four-helix bundle. This complex has two nonidentical HTH motifs and a single ligand-binding domain. The two nonidentical HTHs from each TFR bind within the high-affinity, nonpalindromic operator motif, with Arg53 and Lys262 inserted into the major groove. Site-directed mutagenesis of Arg53 to alanine abrogated DNA binding, validating the Mce3R/DNA structure obtained. Among 811,645 particles, 63% were Mce3R homodimer bound to two duplex oligonucleotides. Mce3R homodimerizes primarily through α15, and each monomer binds to an identical site in the DNA duplex oligonucleotide.

*M. tuberculosis* (*Mtb*), the causative agent of tuberculosis (TB), is the second deadliest infectious disease after the COVID-19 virus.^1^ Throughout its coevolution with the human host, *Mtb* has developed the ability to survive and adapt to the changing environment within the host.^2−4^ However, the precise mechanisms through which *Mtb* controls its gene expression for survival and persistence remain unclear. Transcriptional repressors, including members of the TetR family (TFR), play a crucial role in *Mtb*’s physiological response to varying metabolic environments. TFRs are found in different bacterial species where they regulate genes associated with various activities, such as lipid, carbon, and nitrogen metabolism, antibiotic production, and quorum sensing.^5−10^

The *Mtb* genome has about 52 TFR-encoding genes.^11,12^ Among these, a notable TFR in *Mtb* is Mce3R (Rv1963c), which is encoded by a gene located upstream of the virulence-associated *yrbE3A* (*mce3*) operon and represses genes within the Mce3R regulon.^13^ The genes encoded within the Mce3R regulon play key roles in lipid metabolism, resistance to oxidative stress, and persistence within the host.^11,14−17^ Additionally, *mce3R* is encoded in the Mce3R regulon and is thus autoregulated.^14^ Our study of Mce3R was prompted by its potential involvement in the cholesterol metabolism pathway, as expression of the Mce3R regulon is induced in the presence of cholesterol.^18^ Enzymes encoded within the regulon share a cholesterol pathway-specific β-oxidation motif.^19,20^ Recent discoveries have also highlighted the requirement of Mce3R-regulated genes for 6-azasteroid^21^ activity and 7-phenyl benzoxaborole^22^ antimicrobial activity. Orthologs of *mce3R* are exclusive to a subset of pathogenic mycobacterial species, emphasizing the critical role of this regulon in host persistence. As a result, we initiated an analysis of the structure and function of Mce3R.

Typical TFRs consist of two main domains: an N-terminal DNA-binding domain (DBD) and a large C-terminal ligand-binding domain (LBD) (Figure 1A).^23^ Two identical polypeptides composed of 9 α-helices each dimerize through the formation of a four-helix bundle to form a functional homodimeric repressor. The two helix-turn-helix (HTH) domains are highly conserved and insert into the major groove of a palindromic DNA operator region.^24−26^

**Figure 1:**
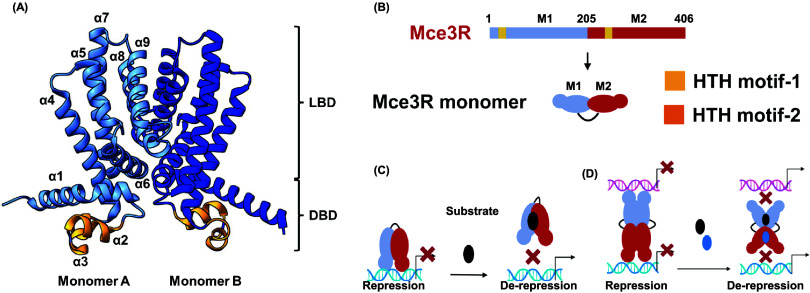
Typical TFR tertiary structure compared to the Mce3R polypeptide sequence. (A) A typical TFR homodimer structure^10^ (TetR-family transcriptional regulator Rha06780 from *Rhodococcus* sp. Rha1, a figure created using PDB 2NX4 coordinates from the Protein Data Bank^30^), in which each protomer is composed of nine α-helices. The ligand-binding domain (LBD, blue) is formed through a four-helix bundle with two helices contributed by each protomer. There are two helix-turn-helix (HTH, orange) motifs, one from each protomer. (B) The primary polypeptide structure of Mce3R illustrates the two TFR fragments: M1 (1–205 amino acids, blue) and M2 (206–406 amino acids, red). There are two HTH motifs (amino acids from 36–55, orange, and 240–259, orange-red) within this single polypeptide. (C) Possible asymmetric assembly of M1 and M2 monomer. (D) Possible symmetric assembly of M1 and M2 as a Mce3R homodimer. Upon binding to the substrate or inducer, the Mce3R will go from a repressed state to a derepressed state, which does not bind to DNA.

Mce3R is a member of a subfamily of double TFR repressors that occurs infrequently in bacterial genomes.^10,27^ In contrast to a typical TFR gene, the *mce3R* gene is a fusion of two distinct TFR sequences that encode a double-TFR polypeptide (Figure 1B).^11,14,27^ Highlighting this feature of Mce3R more than 10 years ago, a study predicted 204 similar, putative double-TFR proteins in *Actinomycetes*.^11,28^ We refer to the N-terminal and C-terminal TFR polypeptides of Mce3R as M1 and M2, respectively. Previous studies have indicated that the M1 and M2 polypeptides of Mce3R are homologous to distinct subgroups within the TFR family, with greater sequence similarity observed among M1 sequences compared to their partner M2 sequence.^10^ However, the tertiary structure of these double TFRs, including the interface for assembly of an active TFR and mode of DNA binding, remains poorly understood.^10,12,29^ The nature of the complex formed determines the number of distinct DNA-binding sites repressed and the number of unique ligands that may derepress Mce3R.

The key question we aimed to address in this study is the tertiary and quaternary structures of the Mce3R protein complex and the DNA repressor complex. We investigated whether each individual Mce3R polypeptide folds into a functional TFR protein that is asymmetric, with respect to the four-helix bundle. This folding pattern would result in a TFR with two nonidentical HTH motifs and an M1–M2 ligand-binding interface. The M1–M2 model would require a single type of ligand to derepress (Figure 1C). Alternatively, two polypeptides could dimerize to form two symmetric yet distinct functional TFRs: an M1M1 TFR and an M2M2 TFR, with two distinct ligand-binding domains and distinct HTH palindromic recognition motifs for each TFR (Figure 1D).

Here, we demonstrate that the M1–M2 asymmetric fold, featuring a single type of ligand-binding domain, is the functional form of Mce3R. The repressor complex binds to an asymmetric DNA operator site. This tertiary complex, in turn, forms a C2-symmetric homodimer. This structure provides entry to understanding how repression and induction may occur in the genomic milieu.

## Materials and Methods

### Materials, Strains, Media, and General Methods

All chemicals and reagents were of analytical reagent grade and were procured from commercial sources. Single-stranded oligonucleotides, both unlabeled and Cy3-labeled at the 5′ end, and nuclease-free duplex buffer were purchased from Integrated DNA Technologies, Inc. (IDT, Coralville, IA). ProTEV Buffer was acquired from Promega (Madison, WI). iProof GC buffer and iProof DNA polymerase were obtained from Bio-Rad Laboratories (Hercules, CA). The DNA Clean & Concentrator kit was purchased from Zymo Research Corporation (Irvine, CA). The TEM data collection, cryo-EM grid preparation, data collection, image processing, model building, and validation were conducted by Biortus Wuxi Co., Ltd.

Buffers used were: Buffer A: 20 mM Tris (pH 7.4), 0.2 mM NaCl, and 0.1 M EDTA; Buffer B: 20 mM Tris (pH 7.4), 0.2 mM NaCl, 10 mM maltose, and 0.1 M EDTA; Buffer C: 20 mM Tris (pH 7.5), 0.2 M NaCl, and 10 mM imidazole; Buffer D: 20 mM Tris (pH 7.5), 0.2 M NaCl, and 500 mM imidazole; Buffer E: 20 mM Tris-HCl (pH 7.5) and 0.2 M NaCl; Buffer F: ProTEV buffer (20×, 1 M HEPES pH 7.0, 10 mM EDTA) with 1 mM DTT. Buffer G: 50 mM HEPES (pH 7.5), 500 mM NaCl, and 5% glycerol; Buffer H: 50 mM HEPES (pH 7.5), 500 mM NaCl, 5% glycerol, and 10 mM maltose; Buffer I: 50 mM HEPES (pH 7.5) and 200 mM NaCl; Buffer J: 25 mM Tris-Base (pH 8.5) and 25 mM NaCl. Buffer K: 25 mM Tris-Base (pH 8.5). Buffer L: 25 mM Tris-Base (pH 8.5) and 1 M NaCl. Buffer M: 10 mM HEPES (pH 7.5), 150 mM KCl, 0.5 mM EDTA, 0.2 DTT, 1 mM MgCl_2_, 12% glycerol. Nuclease-Free Duplex Buffer: 30 mM HEPES (pH 7.5); 100 mM potassium acetate; iProof GC buffer (5×): GC buffer, 7.5 mM MgCl_2_. TBE buffer (5×): 445 mM Tris-Base, 445 mM boric acid, and 0.01 M EDTA.

#### Expression and Purification of Mce3R

All cloning steps were performed in *Escherichia coli* XL1blue. Full-length *mce3R* (Rv1963c) was PCR-amplified from *Mtb* H37Rv with the primers shown in Table S1 and cloned as an N terminus MBP-His6-tagged-fusion protein into a pET His6MBP TEV LIC cloning vector (2M-T, Addgene plasmid #29708). The resulting plasmid (2MT_*mce3R*) was introduced into *E. coli* BL21(DE3) and the transformed *E. coli* was grown in 2x-YT medium containing 100 μg/mL ampicillin and 0.1% glucose at 37 °C until the OD_600_ reached 0.6–0.8, and the culture was induced with 1 mM IPTG (final concentration) at 25 °C for 16 h.

All protein purification steps were conducted at 4 °C unless stated otherwise. Bacterial cells were pelleted at 5180*g* for 15 min. The resulting cell pellet was then resuspended in either Buffer A or Buffer C, followed by cell lysis using a cell disruptor and ultracentrifugation of the suspension at 186,010*g* for 1 h at 4 °C. The purification of MBP-His_6_-Mce3R recombinant protein involved a five-step process. (1) The ultracentrifuged supernatant was loaded onto an amylose resin column, washed with Buffer A, and eluted with Buffer B. Alternatively, for protein preparation on an IMAC His bind resin, the ultracentrifuged supernatant was loaded onto a Ni-NTA column, washed with Buffer C, and eluted with a gradient of Buffer C and Buffer D. (2) Fractions were collected and dialyzed overnight against Buffer C using 30 kDa molecular weight cutoff (MWCO) dialysis tubing. (3) The fusion protein was subjected to TEV protease cleavage in Buffer F for 16 h, with a fusion protein to TEV protease ratio of 8:1 (w/w). (4) Subsequent purification on a Ni-NTA column was conducted using a step gradient of Buffer C and D. Steps were 2X column volumes each: (1) 100% C; (2) 75% C; (3) 50% C; (4) 25% C; (5) 0% C. (5) Size exclusion chromatography (SEC) on a Superdex 200 HiLoad 16:60 column (GE Healthcare Biosciences Corp., Piscataway, NJ) with elution using Buffer E yielded the purified protein.

The purity of Mce3R was assessed on 12% SDS PAGE, and the protein’s identity was confirmed via tryptic digestion followed by MALDI-TOF spectrometry. The approximate molecular weight (MW) of the native-folded Mce3R was determined using a Gel Filtration Calibration Kit containing high and low MW standards: aldolase (160 kDa), conalbumin (76 kDa), ovalbumin (45 kDa), ribonuclease A (13.7 kDa), and aprotinin (6.5 kDa).

For cryo-EM, bacterial cells were lysed in Buffer G and ultracentrifuged at 41,200*g* for 30 min. Then, the supernatant was loaded onto an amylose resin, which was washed with Buffer G and eluted with a gradient of Buffer G and H. Then, the same sequential steps 2–5 were followed, and the final SEC was performed with Buffer I.

#### Expression and Purification of M1 and M2

Gene sequences encoding amino acids 13–193 (M1) or 219–399 (M2) were PCR-amplified from 2MT*_mce3R* with the primers shown in Table S1 and cloned as N terminus His_6_tagged-fusion protein into pET28b. The resulting plasmids (pET28b_M1 and pET28b_M2) were introduced into *E. coli* Rosetta (DE3) pLysS and the transformed *E. coli* was grown in 2X-YT medium containing 50 μg/mL kanamycin and 25 μg/mL chloramphenicol at 37 °C until the OD_600_ reached 0.6–0.8 and the culture was induced with 1 mM IPTG (final concentration) at 37 °C for 3–4 h. Only M1 was expressed, and M2 was not expressed in these conditions as well as in other conditions.

Bacterial cells were pelleted at 5180*g* for 15 min. The resulting cell pellet was then resuspended in Buffer C, followed by cell lysis and ultracentrifugation of the suspension at 186,010 g for 1 h at 4 °C. The ultracentrifuged supernatant was then loaded onto IMAC His bind resin, washed with Buffer C, and eluted with a step gradient of Buffer C and Buffer D, following the same procedure used for Mce3R Ni-NTA purification. Fractions were collected and dialyzed overnight against Buffer J by using 10 kDa MWCO dialysis tubing. Ion exchange chromatography was carried out with a step gradient of Buffers K and L as follows: (1) a 2× column volume wash with 2% Buffer L, (2) a 2× column volume wash with 3% Buffer L, and (3) elution using a gradient of 10–20 column volumes with increasing ionic strength up to 0.5 M NaCl (50% Buffer L). Fractions that contained M1 were collected and concentrated. A final purification step involved size exclusion chromatography (SEC) on a Superdex 200 HiLoad 16:60 column (GE Healthcare Biosciences Corp., Piscataway, NJ) with elution using Buffer E.

#### Mce3R Motif Search Using MEME

A DNA-binding motif search was carried out using MEME (Multiple Em of Motif Elicitation) in the classical mode.^31^ The 200 bp upstream of the *yrbE3A* (*mce3*) promoter in *Mtb*, *Mycobacterium bovis*, and *Mycobacterium marinum* were obtained from Mycobrowser.^12^ The minimum and maximum widths for motifs were set at 6 and 110 nucleotides, respectively.

### Electrophoretic Mobility Shift Assays (EMSA)

All complementary single-stranded DNA (ssDNA) oligonucleotides were dissolved in nuclease-free duplex buffer (IDT). Double-stranded DNA (dsDNA) was prepared through PCR extension. The PCR reaction was conducted in a 50 μL reaction volume with 1× iProof GC buffer, 0.2 mM dNTP, Cy3-labeled forward primer (1.25 μM), reverse primer (1.25 μM), 1.5 μL of DMSO (100%), and 0.02 U/μL iProof DNA polymerase to yield probe A, A′, A*, and B. After PCR extension, each dsDNA product was concentrated using a DNA Clean & concentrator, and the final product was eluted with 12 μL of nuclease-free duplex buffer and stored at −20 °C. Alternatively, for Cy3-labeled Probe C, a heat block was used. The two complementary ssDNA strands were combined in equal molar concentrations, heated to 95 °C for 5 min, allowed to cool to RT, and then stored at −20 °C.

EMSA assays (20 μL) were performed in Buffer M. For *K*_d_ experiments, the Cy3-labeled DNA concentration was kept at 10 nM while Mce3R and M1 protein concentrations were varied from 0–128 nM. Aliquots were incubated for 30 min on ice in the dark and then loaded onto a 2.5% polyacrylamide gel containing 0.5× TBE and 2.5% glycerol. The gels were run at 4 °C and 50 V for 2 h in 0.5× TBE 25% glycerol buffer. Intensities of DNA bands were scanned using a BIO-RAD Chemidoc MP Imaging system. The *K*_d_ data were fitted using eq 1 in GraphPad software, assuming two independent binding sites for the Mce3R dimer. The constants are a, the percentage of maximum bound (fixed at 100); L, the total DNA concentration; *K*, the dissociation constant (*K*_d_, nM); and *X*, protein concentration. All EMSA assays were performed in triplicate.
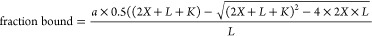
1

#### Grid Preparation

The Mce3R protein (15.8 μM) and Probe A (7.9 μM) duplexes were mixed in a molar ratio of 2:1 and incubated on ice for 1 h. The concentration of the complex was adjusted to 1.0 mg mL^–1^. For grid preparation, a droplet of 3 μL of the Mce3R-DNA complex was applied onto a holey gold foil grid (Quantifoil Micro Tools GmbH; UltrAuFoil Holey Gold, R1.2/1.3, 300 mesh), which was freshly glow-discharged using a glow-discharge cleaning system (PELCO easiGlow), for 100 s at 15 mA. After incubation at 4 °C and 100% relative humidity for 1 min, the grid was then blotted for 4 s under a force of 4, followed by immediate plunge-freezing in liquid ethane using a Vitrobot Mark IV system (Thermo Fisher Scientific). Grids were stored in liquid nitrogen for data acquisition.

#### Cryo-EM Data Collection

The final cryo-EM data set was collected on a Titan Krios G4 transmission electron microscope operated at 300 kV, which was equipped with a Falcon 4i direct electron detector (Thermo Fisher Scientific). Movies were recorded in the EER format using EPU (Thermo Fisher Scientific) at a magnification of 130 K, resulting in a physical pixel size of 0.95 Å. The slit width of the Selectris energy filter (Thermo Fisher Scientific) was set at 10 eV. A defocus range between −1.1 to −2.2 μm was applied for data collection, and the total dose used for data collection was 55 e–/Å^2^. The whole data set consisted of 6913 movie stacks.

#### Cryo-EM Image Processing

Most of the image processing steps were carried out within the CryoSPARC package (v4.2.1)^32^ while some of the processing jobs were performed using Relion (Relion 3.1) or SIRM_RELION (https://github.com/homurachan/SIRM_RELION).^33^ After motion correction and CTF estimation, images were scrutinized based on a set of criteria, resulting in a subset of 6377 good images. A total of 2,233,517 particles were automatically picked using the blob picker in CryoSPARC, and another set of 1,089,762 particles were picked through Topaz. Particles were then extracted with a box size of 360 pixels and binned by a factor of 4 to accelerate the calculations. After 2D classification, particles corresponding to the selected classes were combined and duplicates were removed, resulting in a set of 811,645 particles. Ab initio reconstructions and heterogeneous refinement were further employed to clean up the data set, giving rise to a subset of 509,682 particles exhibiting a molar ratio of Mce3R: DNA = 1:2. The particles in the subset were subjected to further *ab initio* reconstruction and heterogeneous refinement. A good class comprising 148,362 particles at high resolution was obtained. Particles were then re-extracted with a pixel size of 448 pixels, and nonuniform refinement^34^ was then performed to obtain the reconstructed map at a resolution of 2.72 Å based on the gold-standard Fourier shell correlation using the 0.143 criteria.^35^ From this point, further processing was branched into two different directions with C1 and C2 symmetry applied, respectively. For processing with C1 symmetry applied, particles were imported into Relion, and 3D autorefining, postprocessing, and SIRM_Relion were followed. A final map at a resolution of 2.83 Å was obtained. By imposing C2 symmetry, nonuniform refinement and local refinement were executed to further process the map to improve the map quality. Finally, a density map with a resolution of 2.51 Å was generated. Local resolution was estimated within the CryoSPARC package, and a b-factor of −62 Å^2^ was applied to sharpen the map.

#### Model Building and Refinement

The structure of Mce3R was predicted by AlphaFold2 and was docked into the EM density in UCSF Chimera (version 1.14)^36^ software. The model was then manually adjusted in COOT and was real-space refined in Phenix.^37^ Several interactions of adjustment and refinement were performed to improve the quality of the model. The final model was evaluated using MolProbity.^38^

#### Site-Directed Mutagenesis

Primers utilized for mutagenesis are listed in Table S1. The 2MT-Mce3R plasmid was used as the template for the PCR mutagenesis protocol. The QuickChange site-directed mutagenesis kit—Agilent protocol was followed.^39^ We constructed two variants: R53A and K262A. Subsequently, the R53A variant was expressed and purified using the same protocol utilized for wild-type Mce3R. The K262A variant could not be expressed.

## Results and Discussion

### Bioinformatics Analysis of Mce3R Sequence

**Figure 2:**
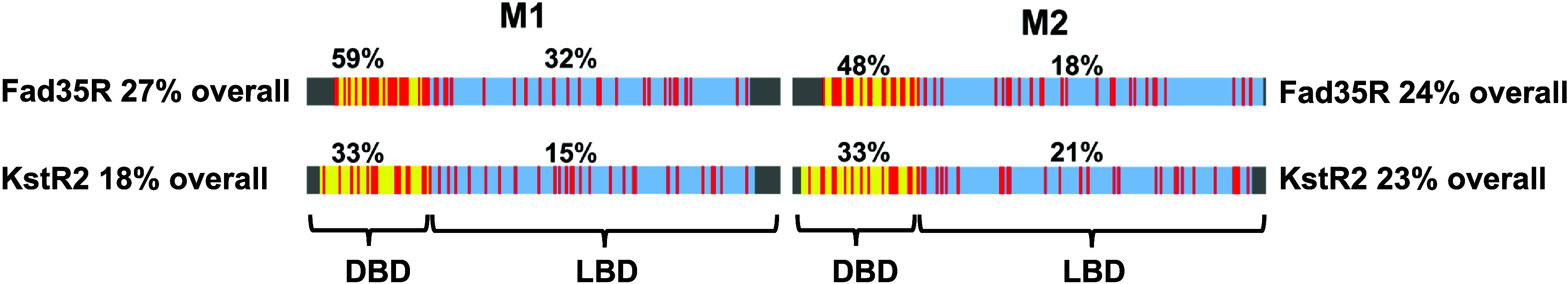
Sequence analysis of Mce3R. Schematic illustration of the Mce3R primary sequence, HTH motifs (yellow, amino acids 36–55 and 240–259). PHYRE2 threading used the M1 and M2 segments separately. Red lines indicate identical amino acids among the indicated proteins. Gray shading indicates no similarity among the indicated proteins. Percentages reported are percentages of amino acid identity. The multiple sequence alignment (MSA) analysis of Fad35R, Mce3R, and M1 or M2 revealed high identities in the DBDs compared to those in the LBDs. DBD amino acid identities: M1/Fad35R, 59%; M2/Fad35R, 48%; M1/KstR2, 33%; M2/KstR2, 33%. LBD amino acid identities: M1/Fad35R, 32%; M2/Fad35R, 18%; M1/KstR2, 15%; M2/KstR2, 21%.

As a first step in understanding more about Mce3R’s role in *Mtb* gene regulation, we undertook a biochemical and biophysical analysis of the regulatory protein sequence. Initial bioinformatics analysis of the full-length Mce3R using PHYRE2 and Swiss-Model revealed that the individual TFR regions of Mce3R are homologous to KstR2 (PDB 4W97) and Fad35R (PDB 4G12) TFRs from *Mtb*.^40−42^ Both are typical TFRs that function as homodimers. We conducted a further analysis using the separate regions of the sequence corresponding to M1 and M2 in threading and alignment to assess each TFR region separately. M1 and M2 share 27 and 24% overall identity, respectively, with Fad35R. M1 and M2 share 18 and 23% overall identity, respectively, with KstR2 (Figure 2).

We further segmented the M1 and M2 sequences and analyzed the sequence identities of their DBD or LBD with the corresponding DBD or LBD of either Fad35R or KstR2. In the multiple sequence alignment (MSA) analysis,^43^ the percentage identity for M1 or M2 was significantly higher in the DBDs compared to the LBDs. Overall, Mce3R shares the highest identity of amino acids with Fad35R, with identical amino acids clustered in the DBDs. Typically, DBDs are more conserved between TFRs.^10^ The lower identities between LBDs suggest that the ligand(s) for Mce3R derepression are distinct from those of Fad35R or KstR2.

### Expression of Mce3R, M1, and M2

Full-length Mce3R was expressed as a maltose-binding protein (MBP) fusion. After purification, MBP was removed by proteolytic cleavage to yield the full-length Mce3R protomer (Figure S1A). Full-length Mce3R has a calculated molecular weight (MW) of 44.3 kDa. Size exclusion chromatography (SEC) purification and analysis revealed that Mce3R elutes as a homodimer (data not shown).

To aid the evaluation of Mce3R dimerization and determine whether the dimer is an M1M2 TFR assembly or an M1M1/M2M2 TFR assembly (Figure 1C,D), we undertook the preparation of the M1 and M2 TFR segments as individual protomers. Monomeric M1 has a calculated MW of 20.2 kDa. During size exclusion chromatography (SEC), M1 eluted as two distinct peaks. The first peak corresponds to the M1 tetramer, and the second peak corresponds to the M2 dimer (Figure S1B). We could not obtain expression of the M2 protein in soluble or insoluble form (data not shown). We assume that the fragment was insufficiently stable in the cell to be prepared.

### DNA-Binding Analysis

Santangelo et al. aimed to identify the DNA-binding motifs of Mce3R through DNase footprint analysis and the utilization of MEME and MAST software.^14^ In their DNase footprint analysis, they discovered two protected regions of approximately 33 bp and 32 bp within the *mce3R-yrbE3A* intergenic region and, through computational analysis, predicted multiple conserved motifs in both the *mce3R-yrbE3A* and Rv1935c-Rv1936 intergenic regions. Here, we focused on the 200 bp upstream of the *yrbE3A* (*mce3*) operon (2,207,477–2,207,699 bp). We identified conserved nucleotide regions among the orthologous Mce3R operator sequences in the *Mtb*, *M. bovis*, and *M. marinum* regions upstream of the *yrbE3A* (*mce3*) orthologous operons,^14^ and used MEME software to predict DNA-binding motifs on the basis of these homologies.^31^

**Figure 3:**
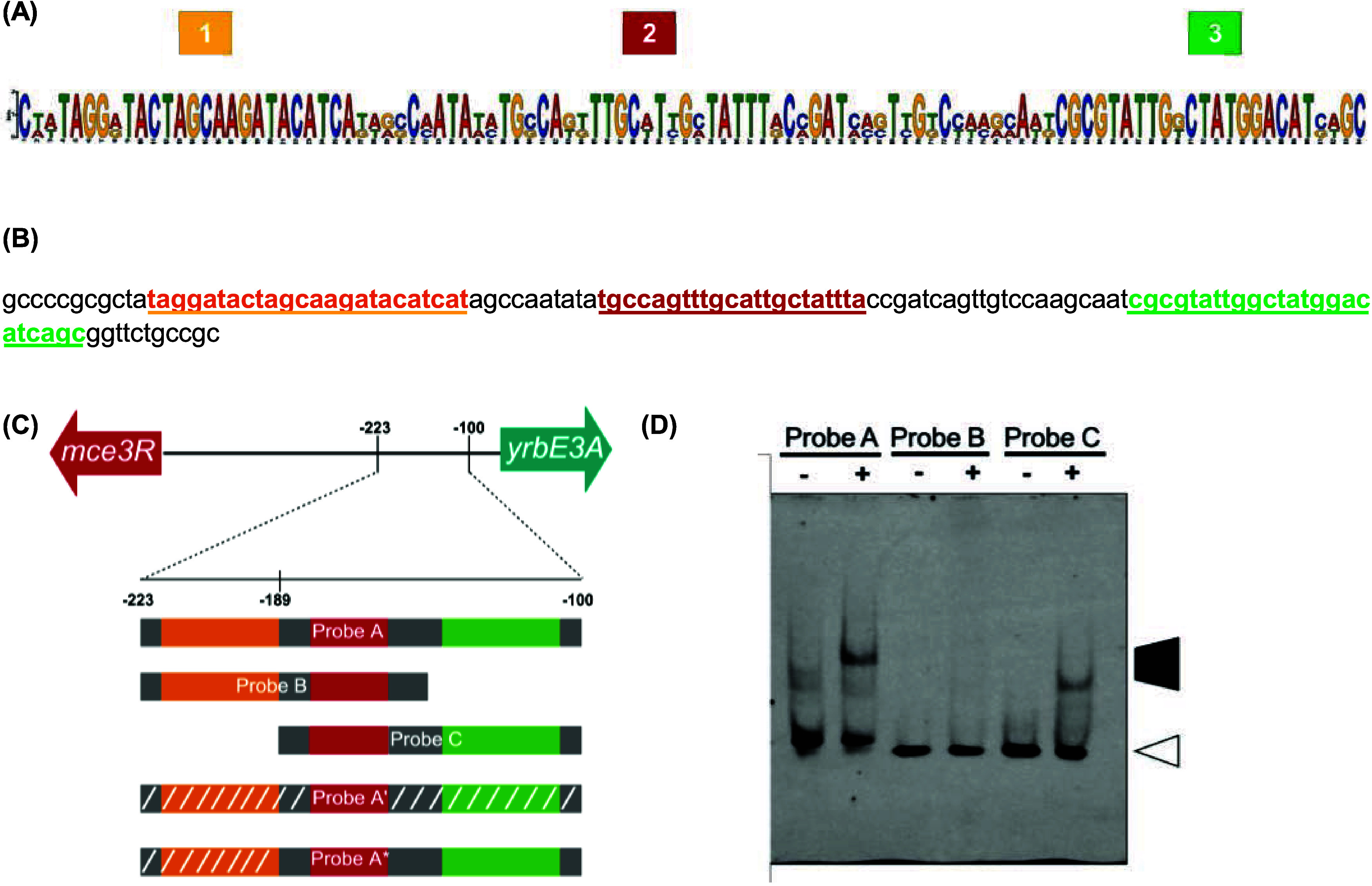
*yrbE3A (mce3)* promoter region exhibits three motifs conserved among orthologues. (A) Sequence motifs identified using the MEME search tool. (B) Probe A sequence with regions 1, 2, and 3 underlined. Yellow, red, and green sequences correspond to regions 1, 2, and 3, respectively. (C) Schematic representations of duplex oligonucleotides synthesized for EMSA experiments. Probe A′ and A* contain scrambled sequences with the same percentage of A, G, C, and T as the corresponding native genomic Probe A sequence. The scrambled segments are indicated by hashing. (D) Fixed concentration EMSA experiments show that Mce3R binds to Probe A and Probe C. Mce3R (8 nM) and Cy3-Probe A, Probe B, or Probe C (10 nM) were analyzed on a 2.5% native polyacrylamide gel. Each binding assay contains 20 ng of polydI-dC. The dark trapezoid indicates the Mce3R-DNA complexes; the white arrow indicates free Cy3-DNA.

The footprint analysis results of Santangelo et al. suggested that the Mce3R binding site is not a short 18–21 bp palindrome sequence, as observed for typical TFRs.^10,14,44,45^ Through MEME analysis, we identified a 104-bp-long DNA sequence containing three conserved regions, each about 25 bp in length. The conserved sequences are asymmetric. The three conserved regions are denoted as 1, 2, and 3 (Figure 3A). Two of the three regions (1 and 3) correspond to the DNA footprint identified by Santangelo.^14^ None of the regions contained any direct palindrome sequences (Figure 3A), and the conserved regions are distinct from the operator sequences of KstR2 and Fad35R.

We designed a 123-bp-long oligonucleotide sequence, including the 104 bp conserved region with the extension of the genomic sequence upstream and downstream of the conserved region (Probe A, Figure 3B,C, and Table S3). To further clarify the relative importance of the three regions, we prepared two more DNA oligonucleotides, Probe B and Probe C, that include regions 1:2 (78 bp) and 2:3 (89 bp), respectively, as well as scrambled versions of Probe A. Each sequence was labeled with Cy3 at the 5′-end for use in electrophoretic mobility shift assays (EMSA) (Figure 3C).

### Competitive Electrophoretic Mobility Shift Assay (EMSA)

**Figure 4:**
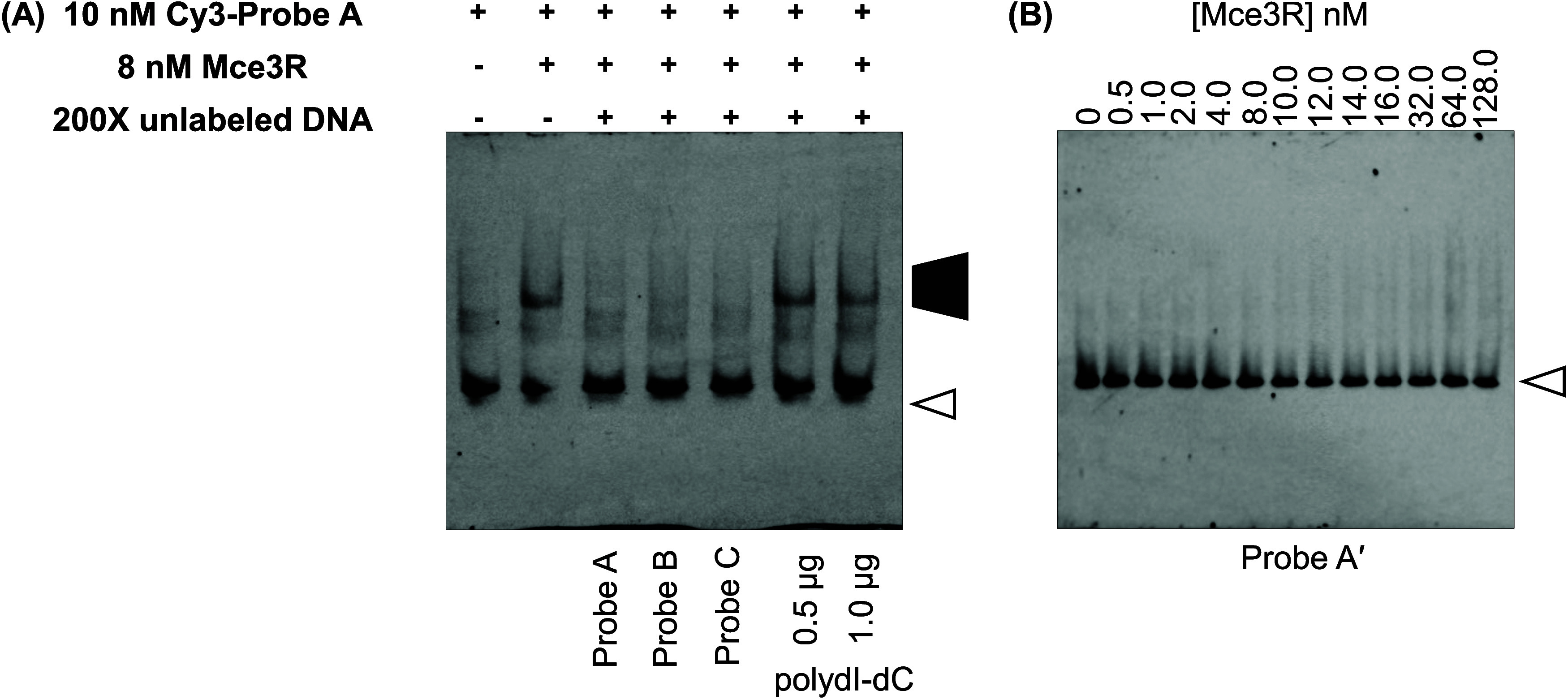
Mce3R-Probe A interaction is specific. (A) Specific and nonspecific competition of protein–DNA interactions. The formation of the retarded mobility complex observed with 8 nM Mce3R (Lane 2) is competitively inhibited upon the addition of a 200-fold excess of unlabeled Probe A, Probe B, or Probe C, but not by the addition of 0.5 or 1.0 μg of the nonspecific polydI-dC. (B) Mce3R does not make a complex with Probe A′ in the presence of 1 μg of polydI-dC. Mce3R concentrations ranged from 0 to 128.0 nM, whereas Cy3-Probe A′ was maintained at a constant concentration of 10 nM. The dark trapezoid indicates the Mce3R-DNA complexes, and the white arrow indicates the free Cy3-DNA-labeled DNA duplex.

The initial EMSA at fixed Mce3R duplex DNA concentrations revealed that Mce3R forms complexes with Probe A or Probe C (Figure 3D), whereas no complex was formed with Probe B. To confirm Cy3 does not alter the specificity of Mce3R binding, we conducted a competition assay using a 200-fold excess of unlabeled Probe A, Probe B, or Probe C. Additionally, we used 0.5 and 1 μg of polydI-dC nonspecific DNA as a nonspecific competitor to eliminate any nonspecific Mce3R-DNA complex formation (Figure 4A). In the presence of a 200-fold excess of unlabeled Probe A, Probe B, or Probe C, the Mce3R-Probe A interaction completely disappeared. This observation suggests that all three unlabeled DNA sequences were able to competitively displace the labeled Probe A, including very high concentrations of Probe B. The presence of nonspecific competitors did not prevent complex formation, indicating that the Mce3R- Probe A interaction is highly specific.

To further investigate the specificity of the Mce3R-Probe A interaction, we tested a 123-bp-long oligonucleotide containing the same nucleotides as Probe A in a scrambled sequence (Probe A′, Figure 3C). As shown in Figure 4B, we did not observe any Mce3R-Probe A′ complex formation.

**Figure 5:**
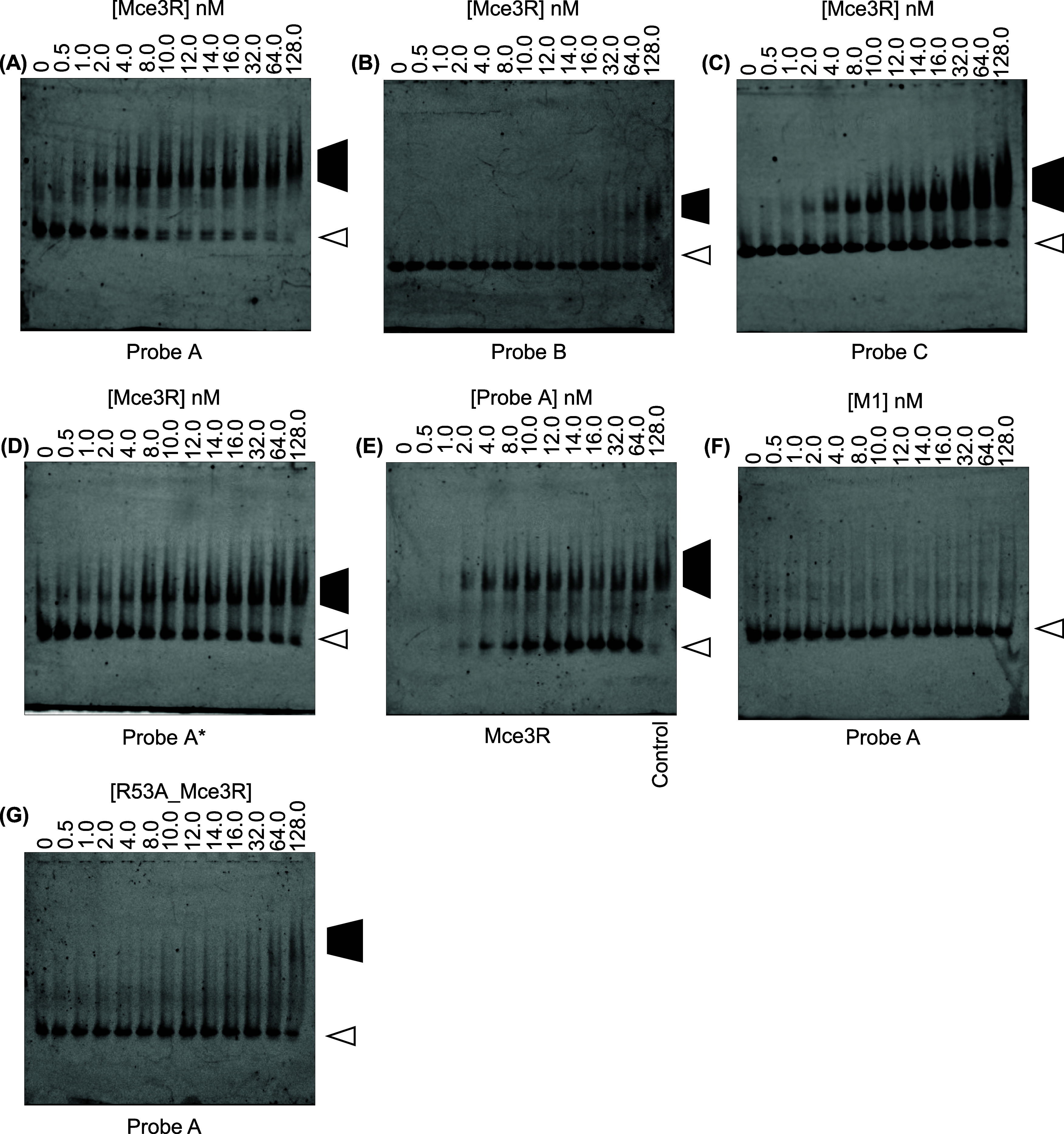
EMSA determination of Mce3R affinity for selected oligonucleotides. (A) Mce3R with Probe A; (B) Mce3R with Probe B; (C) Mce3R with Probe C; and (D) Mce3R with Probe A*. (E) Mce3R was incubated with increasing concentrations of Cy3-labeled Probe A. The control lane contains 30 nM Mce3R and 10 nM Cy3-labeled Probe A. (F) EMSA analysis of M1 with Probe A. (G) EMSA analysis of the R53A_Mce3R mutant binding to Probe A. Each mixture was analyzed on a 2.5% native polyacrylamide gel. The dark trapezoid indicates the Mce3R-DNA complexes and the white arrow indicates the free Cy3-DNA-labeled DNA duplex. Cy3-labeled DNA (10 nM) was incubated with increasing concentrations of (A–D) Mce3R, (F) M1 (G) R53A_Mce3R variant (0–128.0 nM). (E) Mce3R (5 nM) was incubated with increasing concentrations of Cy3-labeled Probe A (0–18 nM). Representative gels are shown. Each experiment was performed in triplicate.

To measure dissociation constants for Mce3R and operator DNA sequences, we held the labeled DNA concentration constant (10 nM), varied the Mce3R concentration from 0 to 128 nM, and analyzed complex formation by EMSA. The *K*_d_ for the Mce3R/Probe A complex is 2.4 nM (Figure 5A). Analogous experiments with Probe B and Probe C showed Mce3R have reduced affinities for these shorter duplexes compared to those of Probe A (Figure 5B,C). No Mce3R/Probe B complex was observed below a concentration of 32 nM Mce3R. The affinity of Mce3R for Probe C was still an order of magnitude weaker than the affinity for Probe A (Table 1).

**Table 1:** *K*_d_ Values of Mce3R with Different DNA Motifs

DNA Motif	*K*_d_ (nM)
probe A	2.4 ± 0.7
probe B	>100
probe C	49 ± 5
probe A*	59 ± 10

Next, we asked whether the different affinities of Probe B and Probe C are due to length differences. Therefore, we tested a 123-bp duplex in which only region 1 was scrambled, Probe A* (Figures 3C and 5D). Mce3R has a lower affinity for Probe A* compared with Probe A. The Mce3R affinities for Probe C and Probe A* are the same within experimental error. Taken together, these data suggest that Mce3R binds to high-affinity region 3 and that secondary binding to region 1 enhances affinity.

Although Mce3R exhibits different affinities for Probe A versus Probe C, both complexes exhibit similar migration patterns. Up to about 32 nM Mce3R, the duplexes form a complex with the same molecular weight, followed by the appearance of a higher molecular weight complex upon increasing the Mce3R concentration. This shift could be either the result of higher-order complexes being formed or indicative of Mce3R aggregation at high concentrations.

To investigate whether different complexes are formed, we conducted EMSA experiments with the Mce3R concentration fixed at 5 nM while varying the Cy3-labeled DNA from 0 to 18 nM (Figure 5E). The results indicate that a single complex is formed up to 18 nM Probe A, whereas, in the control, a higher MW complex is observed. If the limiting species is DNA Probe A, the MW of the complex with Mce3R increases when the Mce3R concentration exceeds 10 nM. Excess Mce3R might result in possible aggregation. By comparing these results, we conclude that the high molecular weight observed may not be due to a higher Mce3R-DNA stoichiometry but rather due to aggregation.

### Cryo-EM Structure of Mce3R/Probe A Complex

**Figure 6:**
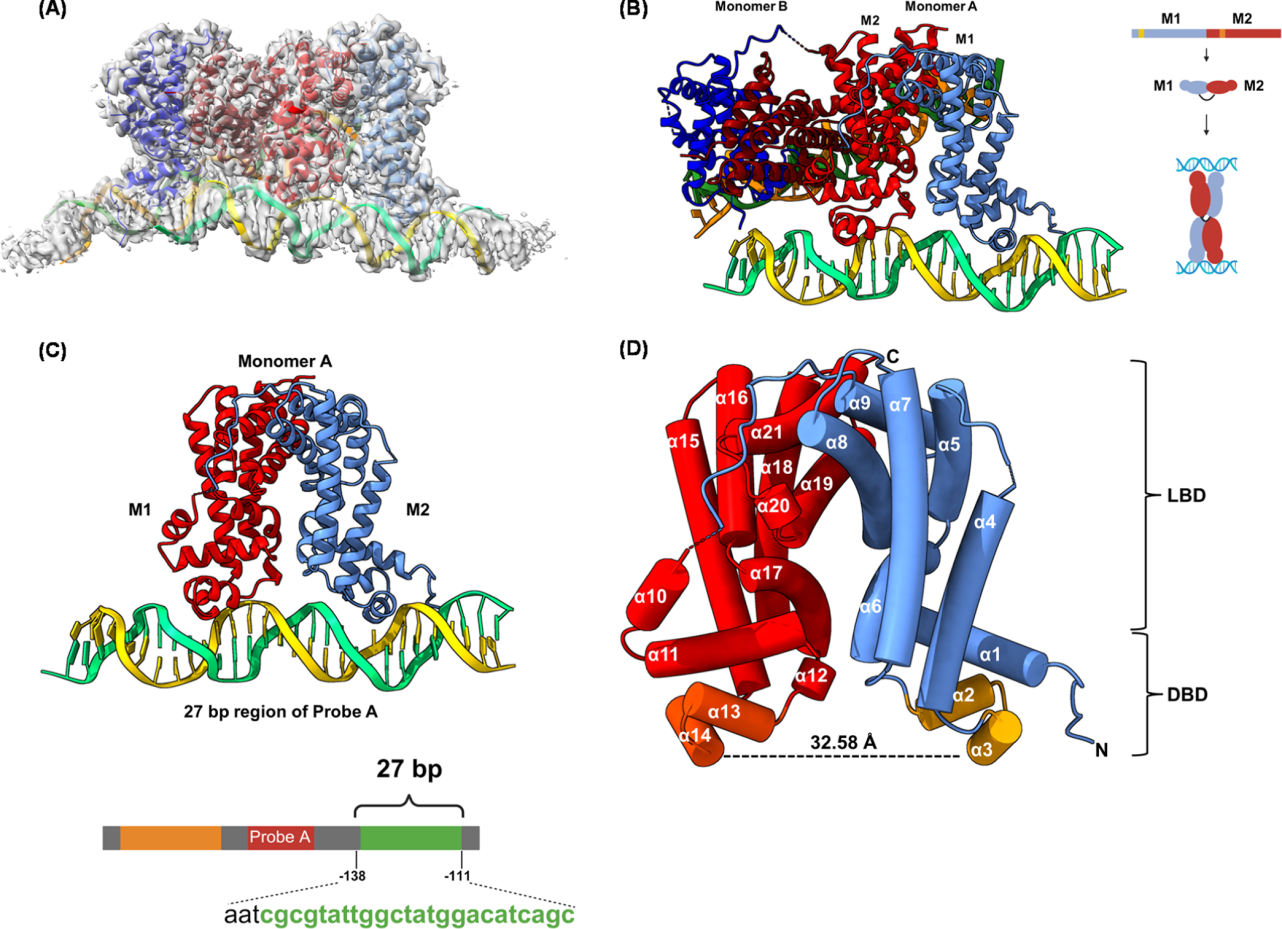
Cryo-EM Structure of Mce3R/Probe A Complex. (A) Mce3R/Probe A cryo-EM density map and the final Mce3R/Probe A model. (B) The Mce3R/Probe A complex displaying M1 and M2 in an asymmetric assembly, forming a homodimer. (C) A single Mce3R monomer oligonucleotide complex. The Mce3R monomer is bound to a 27-bp region of the Probe A DNA. (D) Helix cylinder representation of the Mce3R monomer. M1: helices 1–9, M2: helices 10–18. The distance shown is the span between the middle of the two HTHs.

Mce3R (dimer) was incubated with the Probe A duplex in a molar ratio of 2:1 for 1 h. Grid preparation, data collection, and image processing were performed as described in the Methods and MaterialsSection. Among the 811,645 particles picked from the initial rounds of ab initio reconstruction and heterogeneous refinement in CryoSPARC, approximately 63% of the particles (4 out of 8 classes) exhibited a conformation with an Mce3R dimer:Probe A complex ratio of 1:2, while about 37% of the particles exhibited an Mce3R dimer:Probe A complex ratio of 1:1 (Figure S3). We selected the most abundant conformation and performed further reconstruction and refinement. From this refinement, the most abundant class (approximately 29%) was selected to obtain the final structure of the Mce3R/Probe A complex. The final map for the Mce3R/Probe A complex was reconstructed to a resolution of 2.51 Å (Figures 6A, and S4, and Tables S4, and S5). The overall complex is a C2-symmetric dimer.

The cryo-EM structure of Mce3R/Probe A revealed that the Mce3R dimer binds to two identical duplexes of DNA (Figure 6B). The M1M2 TFR monomer forms a C2-symmetric homodimer, and each protomer binds an oligonucleotide duplex. Each polypeptide forms an asymmetric M1M2 TFR with one ligand-binding domain as well as two HTH recognition motifs (Figure 6C). In agreement with the dissociation constants measured by EMSA, Mce3R is bound to the 27-bp region (region 3) of the Probe A duplex. There is no electron density for regions 1 and 2 in the model (Figure 6A).

Each 392-amino acid Mce3R monomer is folded into 21 α-helices (α1-α21) (Figure 6C,D), and each monomer adopts an omega, Ω, shaped TTR-family fold.^42^ A four-helix bundle formed from α-helices α8, α9, α19, and α21 forms the apex of the Ω. One arm of the Ω is formed by M1 and the other arm by M2, resulting in an asymmetric TFR assembly (Figures 1C and 6D). Thus, the four-helix bundle at the core is assembled from the same protomer, unlike typical TFRs in which each protomer contributes two identical helices.^10^

#### DNA-Binding Domains

Consistent with other TFRs, the DBD has two helix-turn-helix (HTH) motifs that bind to the DNA operator. These two HTH motifs are present in a single protomer with an M1 HTH (residues 36–55) and an M2 HTH (residues 240–259). The DNA-binding domain (DBD) of the M1 region of the Mce3R monomer comprises approximately the first 54 residues, although the N-terminal region up to the 14th residue is disordered in the structure. The DBD of the M2 region of the Mce3R monomer comprises approximately 39 residues. In M1, the two DNA recognition helices α2 and α3, and in M2, helices α13 and α14, form HTH motifs that pack against the N-terminal helices α1 and α11, respectively, and stabilize the structure. Helices α3 and α14 directly contact the DNA-binding sites.

DNA-bound TFR structures have restricted conformational freedom, and the HTH domains are separated by an average of 32 to 38 Å in the repressed/DNA-bound state.^42,46,47^ To corroborate that the Mce3R/Probe A structure represents a repressed state, we measured the distance between A48 and P253, residues in α3 and α14, respectively, of the HTHs in the DBD. The distance between these two residues is approximately 32 Å (Figure 6D), and they fit into the major grooves of Probe A that are approximately 35 Å apart. These findings clearly indicate that each Ω-fold monomer behaves similarly to a typical TFR with respect to DNA binding.

**Figure 7:**
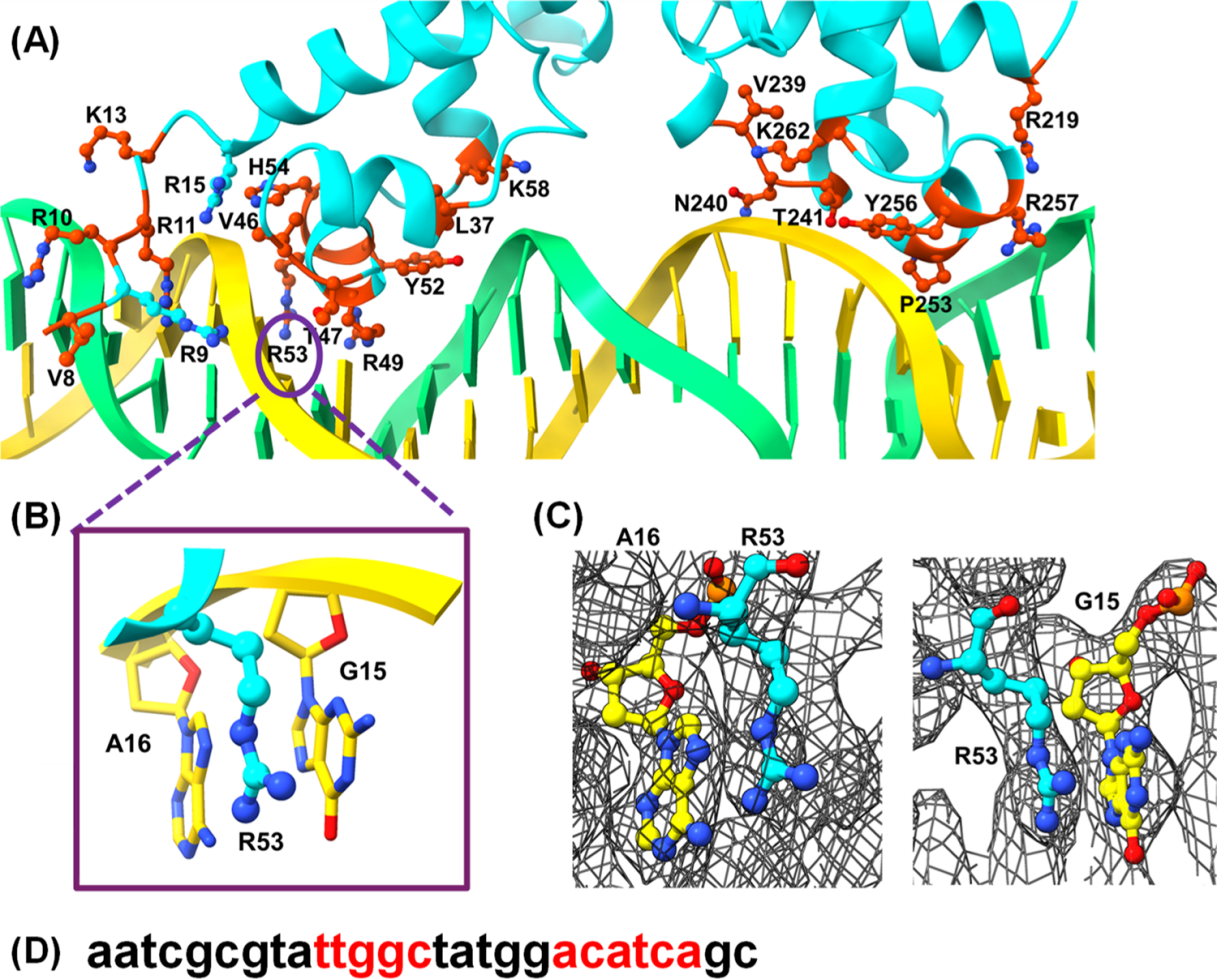
Analysis of the DBD of the Mce3R/Probe A complex. (A) Insertion of the HTH motifs of Mce3R monomer in the 27-bp region of Probe A. Close-up view (B) of R53 in the major groove and (C) the corresponding electron density maps. (D) The nucleotides in the major groove that bind to the HTH motifs of Mce3R are shown in red.

To explore the interaction between amino acids and the Probe A DNA oligonucleotide, we analyzed HTH amino acids within 3.5 Å distance of Probe A. Many of the DNA-binding residues are conserved (T47, R49, Y52, R53, H54, K58, V239, Y256, K262) with other TFRs, including both mycobacterial and nonmycobacterial variants, consistent with the maintenance of the HTH motif structure. In addition, residues R9, R10, R11, R15, A43, V46, R219, R257, T241, and P253 (Figure 7A) are conserved in orthologues of Mce3R in *M. bovis* and *M. marinum*.^11^ We identified Mce3R residues in the proximity of the major groove of the duplex DNA in the cryo-EM structure. There are several positively charged residues in the M1 HTH that interact with the duplex DNA: R10, R11, K13, R49, K52, R53, H54, and K58, and several in the M2 HTH: R219, K262, and R257.

Only a few TFR structures bound to DNA are available, and among them, the mechanism by which TFRs recognize their DNA regulators is varied.^48,49^ For example, the DesT DNA operator has phosphate-backbone contacts with DesT, whereas TetR makes hydrogen bonds with nucleotide bases.^42,50^ Mce3R forms hydrogen bonds with the phosphate backbone and has numerous van der Waals interactions with both the phosphate backbone and the DNA bases. We note that in this study, we have focused on the binding of Mce3R to the *yrbE3A* (*mce3)* promoter site, and Mce3R may form different DNA binding contacts with the operators for the three additional operator sites in the Mce3R regulon.

#### Validation of M1 Binding in the Cryo-EM Structure

The Mce3R structure that we have elucidated represents the repressed state. To probe further whether the Mce3R monomer Ω fold observed in the cryo-EM structure is the functional assembly, we analyzed M1 homotetramer binding to Probe A by EMSA (Figure 5F). No complex was observed between the M1 homotetramer and Probe A at concentrations up to 128 nM Probe A. The inability of the M1 homotetramer to form a complex with the operator sequence is consistent with the absence of palindromic sequences in the intergenic region.

After examination of the structure and the charged residues in the M1 and M2 HTH motifs, which are inserted into the major groove, we selected an arginine and a lysine for mutagenesis studies to assess the validity of the structure. We mutated R53 from the M1 site and K262 from the M2 site. The R53A variant was stable and folded and was purified as a dimer and tetramer. However, the K262A variant could not be produced because the mutation was toxic to bacterial growth. EMSA analysis of the R53A variant clearly illustrated that high-affinity DNA binding is lost upon removal of the single guanidium group from Mce3R (Figure 5G). In the Mce3R/Probe A cryo-EM structure, R53 has four van der Waals contacts with the aromatic bases in the major groove of the DNA (Figure 7B,C) that must stabilize the complex. Taken together with the M1 homodimer assays, the data suggest that Mce3R folds into a single-chain TFR in an Ω fold, and both the M1 and M2 HTH are required for effective binding of Mce3R to the major groove.

#### Dimerization Interface

**Figure 8:**
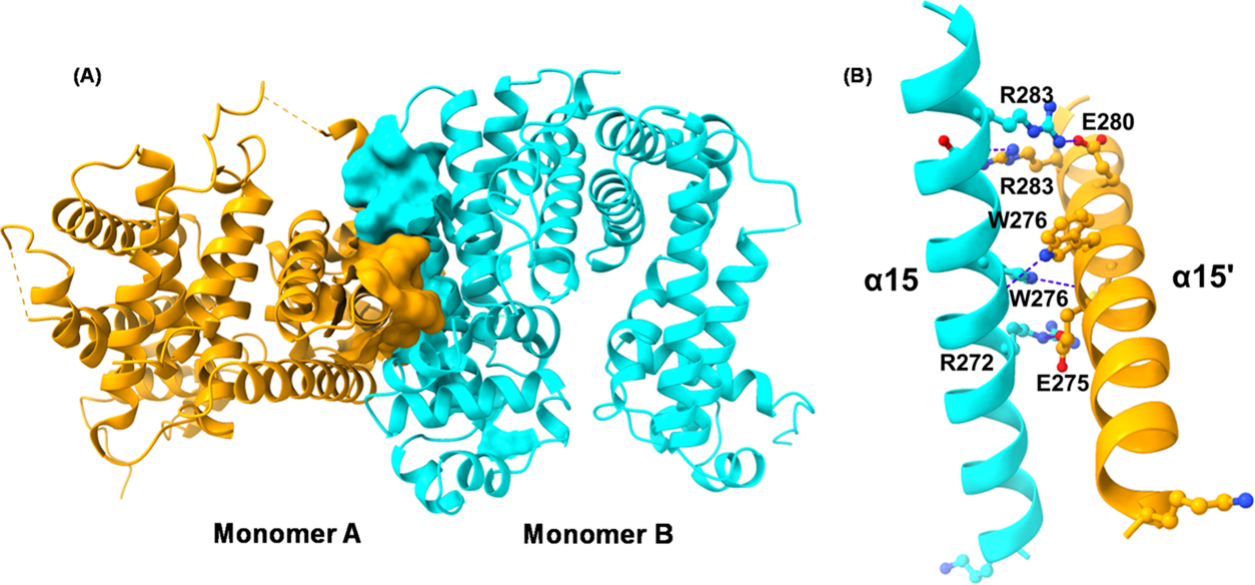
Mce3R dimerization interface. (A) Mce3R dimerization interface comprised of α-helix-15 from each monomer. The buried surface between the two helices is shown. (B) A close-up view of the dimerization interface. Residues in stabilizing interactions are shown in ball and stick style.

The C-terminal domain of the Mce3R monomer consists of 15 α helices (α4-α10 and α15-α21). This domain includes the dimerization interface. The primary dimerization units are helices α15 and α15′ from each monomer. The buried surface area between the two α15 helices measures 500.64 Å^2^ (Figure 8A); the two α15 helices are arranged in a crisscross manner on top of each other. This arrangement is stabilized by approximately 7 hydrogen bonds and 4 salt bridges (Figure 8B). Key residues involved in these interactions include R272, W276, and R283 from monomer A and W276′, C278′, and R283′ from monomer B.

#### Ligand-Binding Domain

**Figure 9:**
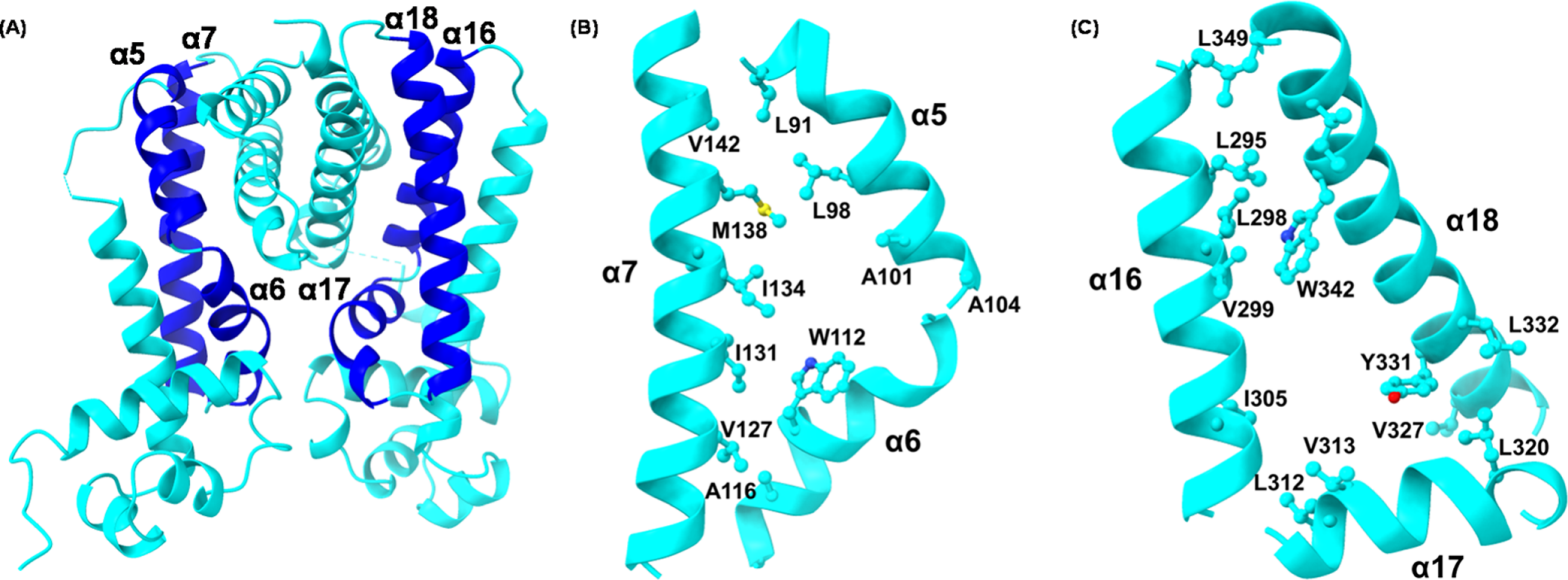
Cartoon view of ligand-binding pocket. (A) Mce3R monomer with the two sides of the ligand-binding pocket highlighted in dark blue. (B) The M1 face of the binding pocket is composed of α5-α7. (C) The M2 face of the binding pocket is composed of α16-α18. Hydrophobic residues aligned in the binding cavity are shown.

The canonical TFR features a ligand or inducer held within a central triangle, which is composed of α5, α6, and α7 from each monomer. In Mce3R, the central triangle is formed by these three helices from M1 and the corresponding three helices from M2: α16, α17, and α18 (Figure 9A). The ligand-binding pocket is hydrophobic, with the α5-α7 side of the pocket lined by hydrophobic and aromatic residues, including V42, L98, A101, A102, A104, W112, A116, V127, I131, I134, V135, and M138 (Figure 9B). The α16-α18 sides of the pocket contain A291, L295, L298, L312, L320, L327, V313, V327, I346, I305, V299, W342, and Y331 (Figure 9C). The lack of charged residues and highly aliphatic hydrophobic nature of the residues lining both sides of the pocket suggests that the inducer may be a fatty acid or steroid derivative consistent with the homologies of other proteins in the Mce3R regulon to fatty acid metabolizing enzymes. The amino acid residues interacting with the CoA moiety of palmitoyl-CoA^42^ and HIP-CoA,^41^ in Fad35R and KstR2, respectively, are only partially conserved in Mce3R. Moreover, the binding orientations of CoA ligands are not strictly conserved between TetR family members, and different residues may contribute to stabilizing binding of the CoA moiety, making it difficult to ascertain whether the Mce3R ligand is a CoA metabolite.

The cavity volume obtained for the monomer using CASTp server^51^ is approximately 1154 Å^3^. Because the structure is in the repressed form, the pocket size is expected to expand upon derepression. The cavity volume for the DesT palmitoyl-CoA site (*Pseudomonas aeruginosa*, homologous to Fad35R) is 790 Å^3^ (for the dimer). The cavity volume for the KstR2 HIP-CoA site is 468 Å^3^ (for the dimer).^52^ This indicates that the Mce3R monomer can accommodate a larger inducer compared to other available TetR-ligand-bound structures or could accommodate two larger ligands within the same cavity.

#### mce3R Resistance Mutants

**Figure 10:**
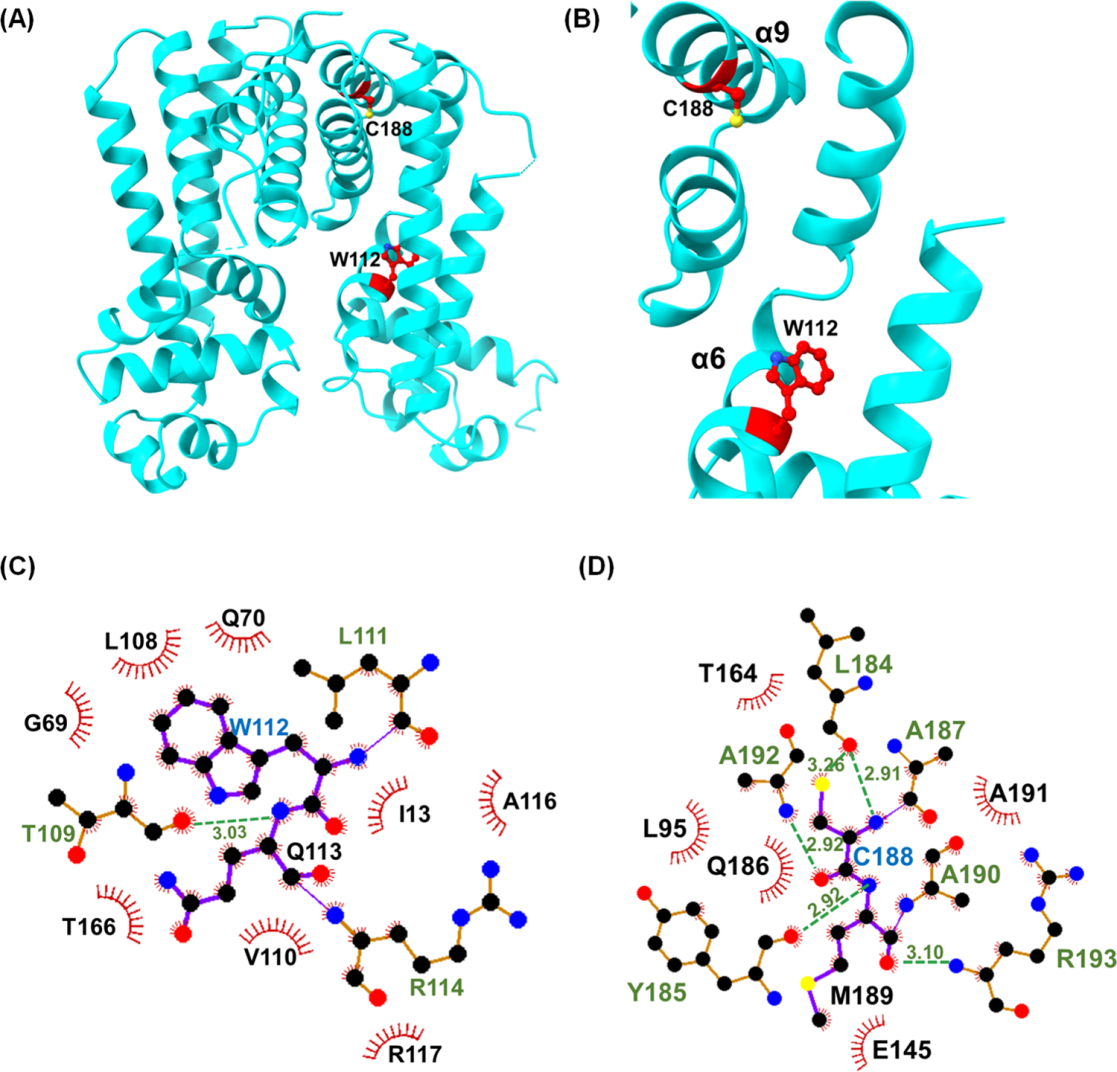
Location of resistance mutations generated in mce3R upon treatment with 7-phenyl benzoxaboroles. (A) Location of W112 and C188 in the Mce3R monomer. (B) Close-up view of W112 and C188 located in α6 and α9 helices, respectively. H bonds and van der Waals interactions between (C) W112 and (D) C188 and residues within 3.5 and 4.5 Å, respectively. Mutated residues were reported by Korkegian et al.^22^

7-Phenyl benzoxaboroles are a novel series of compounds distinct from earlier benzoxaborole series that target leucyl-tRNA synthetase (LeuRS).^22^ Based on *in vitro* resistance studies, the 7-phenyl benzoxaboroles are believed to target NADH dehydrogenase (Ndh), which transfers electrons from NADH to menaquinone and is part of the electron transport chain for ATP synthesis.^53^ The resistance studies also identified point and deletion mutations within the *mce3R* gene.^22^ Both point mutations, W112R and C188F, map to the M1 moiety. W112 is in helix α6, which lines the ligand-binding cavity. This helix is involved in the conformational changes that occur upon ligand binding in TetR proteins. Substitution of tryptophan with an arginine inserts a positive charge into the hydrophobic interface with α7 and α4 helices that would likely cause a shift of these helices and expand the intra HTH spacing and result in depression of Mce3R in the absence of ligand (Figure 10A–C). C188 is in the middle of the α9 helix, where it contributes to packing in the hydrophobic core of the four α helix bundles (Figure 10A,B,D). Replacement of cysteine with phenylalanine would result in an adjustment of the packing in the helix bundle and potentially stabilize the derepressed conformation of Mce3R in the absence of ligand. The location of these mutations suggests that Mce3R mutant resistance to the 7-phenyl benzoxaboroles may derive from constitutive upregulation of the Mce3R regulon to resist intracellular oxidative stress that is generated by inhibition of Ndh and the electron transport chain.

### Summary

To date, there has been no structural information available for duplicated TFR-containing TFRs. Here, we report the first three-dimensional structure of a double TFR repressor that folds as an asymmetric Ω and engages with the DNA major groove of an equally asymmetric operator sequence. The distance between the two DNA recognition sites in each monomer is approximately 32 Å, aligning closely with the major grooves of the DNA. This proximity suggests the compatibility of the two helix-turn-helix (HTH) motifs, enabling them to bind to DNA effectively in the repressed state. The operator region comprises two 25-bp sequences, separated by 53 bp, with a higher affinity observed for Mce3R binding to the downstream site −100 bp upstream of the start codon.

Although the reason for the higher affinity for the downstream (Probe C) region compared to the upstream (Probe B) intergenic region remains unknown, both regions are protected in footprinting experiments^14^ and are conserved between orthologues. One possibility is that weak binding to region 1 conditions strong, highly cooperative binding to region 3. We note that the probes used in this study are too short to loop between the HTH binding motifs on the two protomers of the Mce3R homodimer.^54,55^ The absence of DNA complex formation with M1 or Mce3R-R35A emphasizes the necessity of both the M1 and M2 domains for binding DNA and highlights the asymmetry of this TFR fold. The Mce3R Ω TFR forms a homodimer with both sets of helix-turn-helix (HTH) motifs binding to the same DNA duplex sequence. This quaternary structure suggests that in the genomic context looping of DNA between operator sites in the Mce3R regulon may occur. It will be interesting to investigate whether looping involves other DNA-binding sites within the Mce3R regulon.

Previous work strongly suggests that Mce3R is involved in drug-induced stress-resistant pathways.^21^ The *mce3R* knockout mutant *Mtb* strain exhibits increased resistance to oxidative and lysosomal stress when compared with the wild type. This mutant strain also exhibits an increase in the formation of antibiotic persisters, indicating Mce3R’s role in regulating stress resistance gene expression and its significance for persistence.^11,21^ This role is further corroborated by the elicitation of 7-phenyl benzoxaborole resistance through mutation of Mce3R. Furthermore, Mce3R plays a critical role as a regulator in coordinating responses to environmental changes such as acidic pH, the presence of cholesterol, and low concentrations of K^+^ signals.^27^ The unusual repressor structure presented in this work highlights possible unique avenues by which Mce3R may regulate gene expression to aid in *Mtb* survival and persistence in the host.

The combination of the EMSA method and the high-resolution structure of the DNA-Mce3R complex presented herein provides a toolkit for studying Mce3R regulation. EMSA screening will identify small synthesized molecules as well as native metabolites that induce the Mce3R regulon and possibly compounds that stabilize the repressed complex. The high-resolution structure provides a platform to develop these molecules, synthesize them, design mutagenesis studies, and interpret their binding. Further investigation will yield insights into how the *Mtb* Mce3R stress resistance pathway is regulated and may impact drug resistance.

## Supporting Information Available

Details regarding the protein purification, oligonucleotides used in EMSA, cloning and site-directed mutagenesis, cryo-EM data processing summary, model quality, blotting parameters, and data collection statistics and validations described in the text (PDF)

## Dedication

^†^ Dedicated to Professor Iwao Ojima on the occasion of his 80th birthday on June fifth, 2025.
