# Supplementary material for: Mycobacterium tuberculosis Mce3R TetR-like Repressor Forms an Asymmetric Four-Helix Bundle and Binds a Nonpalindrome Sequence†
Source: ACS Chem Biol. 2024 Nov 15;19(12):2580–92. doi: 10.1021/acschembio.4c00687 (PMC11667970; doi:10.1021/acschembio.4c00687)
Supplement: Supplementary file 1 — cb4c00687_si_001.pdf [file cb4c00687_si_001.pdf]

Mycobacterium tuberculosis Mce3R TetR-like Repressor Forms an Asymmetric Four-Helix Bundle and Binds a Non-Palindrome Sequence

Navanjalee T. Panagoda,[1] Gábor Balázsi,[2,3] and Nicole S. Sampson[1,4,*]

[1]Department of Chemistry, Stony Brook University, Stony Brook, New York, 11794-3400 USA

[2]The Louis and Beatrice Laufer Center for Physical and Quantitative Biology, Stony Brook University, Stony Brook, New York, 11794-5252 USA

[3]Department of Biomedical Engineering, Stony Brook University, Stony Brook, New York, 11794-2581 USA

[4]Department of Chemistry, University of Rochester, Rochester, NY 14627-0216 USA

ORCIDs

NTP: 0000-0003-2366-075X

GB: 0000-0002-6865-5818

NSS: 0000-0002-2835-7760

TABLE OF CONTENTS

Table S1: Oligonucleotide Sequences used for cloning and site-directed mutagenesis. 3

Table S2: Primers used for preparation of Probe A-C, Probe A′ and Probe A*. 4

Table S3: Probes used for EMSA and in Cryo-EM. 5

Table S4: Sample information, blotting Parameters and data collection statistics. 6

Table S5: Model refinement and validation statistics of Mce3R/Probe A complex...................... 7

Figure S1: Purity of Mce3R, M1 and M2 after size exclusion chromatography (SEC). 8

Figure S2: Percentage fraction bound vs Mce3R concentration.. 9

Figure S3: Cryo-EM data processing summary... 10

Figure S4: Map and Model quality of Mce3R (Dimer) Binding to Probe A ds-DNA 11

Table S1: Oligonucleotide Sequences used for cloning and site-directed mutagenesis.

Primer Number	Purpose	Name	Sequence (5'-3')
1	*Mce3R cloning into 2MT (N term-His-MBP)*	Mce3R_For	tacttccaatccaatgcaatggcatccgtcgccc
2		Mce3R_Rev	ttatccacttccaatgttattactatgcgcgcaagagg
3	*M1 cloning into pET28b(N term-His)*	M1_For	atatatgaattcatgaaggaccggaagaagcagat
4		M1_Rev	atatatctcgagtcaccttgccgcggccata
5	*M2 cloning into THST(N term-His-Sumo)*	M2_For	atatatgaattcatgcgctacgagaccctgctggc
6		M2_Rev	atatatctcgagtcacccgctggtcaggatcgcca
7	*Site directed mutagenesis for Mce3R*	R53A For	ttgtatgcacactacgacaacaagcaggcg
8		R53A Rev	gtagtgtgcatacaacgcgcgcgcggtcac
9		R49A_R53A For	accgcggcagcgttgtatgcacactacgac
10		R49A_R53A Rev	caacgctgccgcggtcaccccggccgcctc
11		R53A_N240A For	gccgtcgcaaccagcgaaatcggcaaggga
12		R53A_N240A Rev	gctggttgcgacggccggatagccctgcgc
13		R53A_K262A For	tcttccgcacaggccatcctggacgcgctc
14		R53A_K262A Rev	ggcctgtgcggaagaaaacgaacggtacag

Table S2: Primers used for preparation of Probe A-C, Probe A′ and Probe A*.

Primer Number	Motif	Sequence 5’→3’
15	Probe A For	gccccgcgctataggatactagcaagatacatcatagccaatatatgccagtttgcattgctatttaccgatcagttgtccaagcaatcg
16	Probe A Rev	gcggcagaaccgctgatgtccatagccaatacgcgattgcttggacaactgatcggtaaatagcaatgcaaactggcatatattggctatgatgtatcttgctagtatcctatagcgcggggc
17	Probe B For	gccccgcgctataggatactagcaagatacatca
18	Probe B Rev	caactgatcggtaaatagcaatgcaaactggcatatattggctatgatgtatcttgctagtatcctatagcgcggggc
19	Probe C For	tagccaatatatgccagtttgcattgctatttaccgatcagttgtccaagcaatcgcgtattggctatggacatcagcggttctgccgc
20	Probe C Rev	gcggcagaaccgctgatgtccatagccaatacgcgattgcttggacaactgatcggtaaatagcaatgcaaactg gcatatattggcta
21	Probe A′ For	ccagtgcgacctaaccaaggacctacagat
22	Probe A′ Rev	aggtgcgataactaatataggctcaagtttagcaggtcataaagagaacttgcgaccgtcttctgaggccaacacgattctctcacggacagaatctgtaggtccttggttaggtcgcactgg
23	Probe A* For	ctacaagtgctggaggtcaaatcccgcatcaaca
24	Probe A* Rev	gcggcagaaccgctgatgtccatagccaatacgcgattgcttggacaactgatcggtaaatagcaatgcaaactggcatatattggctatgttgatgcgggatttgacctccagcacttgtag

Table S3: Probes used for EMSA and in Cryo-EM.

Genome Position (bp)	Probe	Sequence 5'→3'
2,207,477 -2,207,600	A	gccccgcgctataggatactagcaagatacatcatagccaatatatgccagtttgcattgctatttaccgatcagttgtccaagcaatcgcgtattggctatggacatcagcggttctgccgc
2,207,477-2,207,555	B	gccccgcgctataggatactagcaagatacatcatagccaatatatgccagtttgcattgctatttaccgatcagttg
2,207,511-2,207,600	C	tagccaatatatgccagtttgcattgctatttaccgatcagttgtccaagcaatcgcgtattggctatggacatcagcggttctgccgc
N/A	A′	ccagtgcgacctaaccaaggacctacagattctgtccgtgagagaatcgtgttggcctcagaagacggtcgcaagttctctttatgacctgctaaacttgagcctatattagttatcgcacct
N/A	A*	ctacaagtgctggaggtcaaatcccgcatcaacatagccaatatatgccagtttgcattgctatttaccgatcagttgtccaagcaatcgcgtattggctatggacatcagcggttctgccgc

Table S4: Sample information, blotting Parameters and data collection statistics.

Sample:	Mce3R/Probe A (molar ratio of Mce3R:Probe A=2:1)
Buffer	50mM HEPES (pH 7.5), 200mM NaCl
Protein batch:	BP32691-20230719
Grid type:	QuantiFoil ultra Au/300； 1.2/1.3
Concentration	1.4 mg/ml
Vol/grid:	3 uL
Blot time:	4 s
Temperature.:	4 ℃
Humidity:	100%
Blot force:	0
Glow discharge:	15mA,100s
Incubation time:	25s
Scope & HT:	Titan Krios 2, 300 kV
Data collection software:	EPU
Detector mode:	counting bin1
Camera:	Krios--Falcon4
Magnification:	130K
Pix size:	0.95 A/pix (physical)
Cs value:	2.7 mm
Number of movies:	6,913
High magnification images:	6,377
Total dose:	55 e-/A^2
Frame number:	40
Frame rate:	1.375 e-/A^2
Defocus range:	-1.1~-2.2μm，0.1μm spacing
Tilt：	0

Table S5: Model refinement and validation statistics of Mce3R/Probe A complex

**Reconstruction**	
Refined particles (no.)	135,814
Final particles (no.)	135,814
Point-group or helical symmetry parameters	C2
Resolution (FSC 0.143, Å)	2.51
Resolution range (local, Å)	
Map sharpening B factor ($Å^2$)	-62
Map sharpening methods	cryoSPARC v4.2.1
**Model composition**	
Protein	Mce3R
DNA	Probe A
**Model Refinement**	
Refinement package	Phenix
Model-Map CC	0.84
Model resolution (Å)	2.8
FSC threshold	0.5
Chains	6
Atoms	8399
Protein residues	763
Nucleotide	124
Water	0
R.m.s. deviations from ideal values	
Bond lengths (Å)(# > 4σ)	0.004
Bond angles (°) (# > 4σ)	0.600
**Validation**	
MolProbity score	1.41
CaBLAM outliers(%)	0.54
Clashscore	7.49
Rotamer outliers (%)	0.50
C-beta deviations	0.00
Ramachandran plot(%)	
Favored	98.67
Allowed	1.33
Outliers	0.00

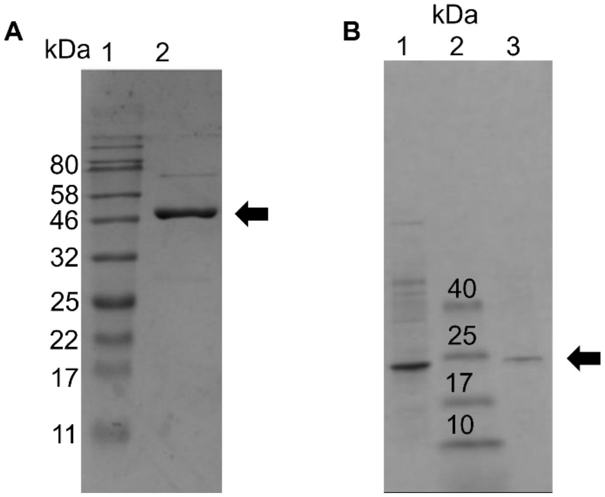

Figure S1: Purity of Mce3R, M1 and M2 after size exclusion chromatography (SEC). (A) Mce3R analyzed on 12% SDS-PAGE after SEC purification. Lane 1, Ladder; Lane 2, Purified Mce3R. (B) M1 analyzed on 4–20% Mini-PROTEAN® TGX™ Precast Protein SDS-PAGE after SEC purification. Lane 1, High molecular weight (MW) eluting fraction from SEC; Lane 2, Spectra™ Multicolor Low Range Protein Ladder; Lane 3, Low MW eluting fraction from SEC.

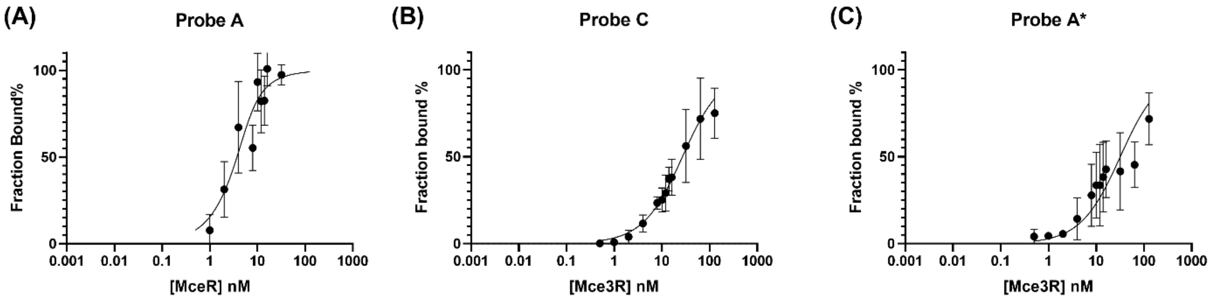

Figure S2: Percentage fraction bound vs Mce3R concentration. (A) Mce3R with Probe A (B) Mce3R with Probe C (C) Mce3R with Probe A*. After EMSA, Intensities of DNA bands were scanned using a BIO-RAD ChemidocTM MP Imaging system. The K_d data were fitted using equation 1 in GraphPad software assuming two independent binding sites for Mce3R dimer.

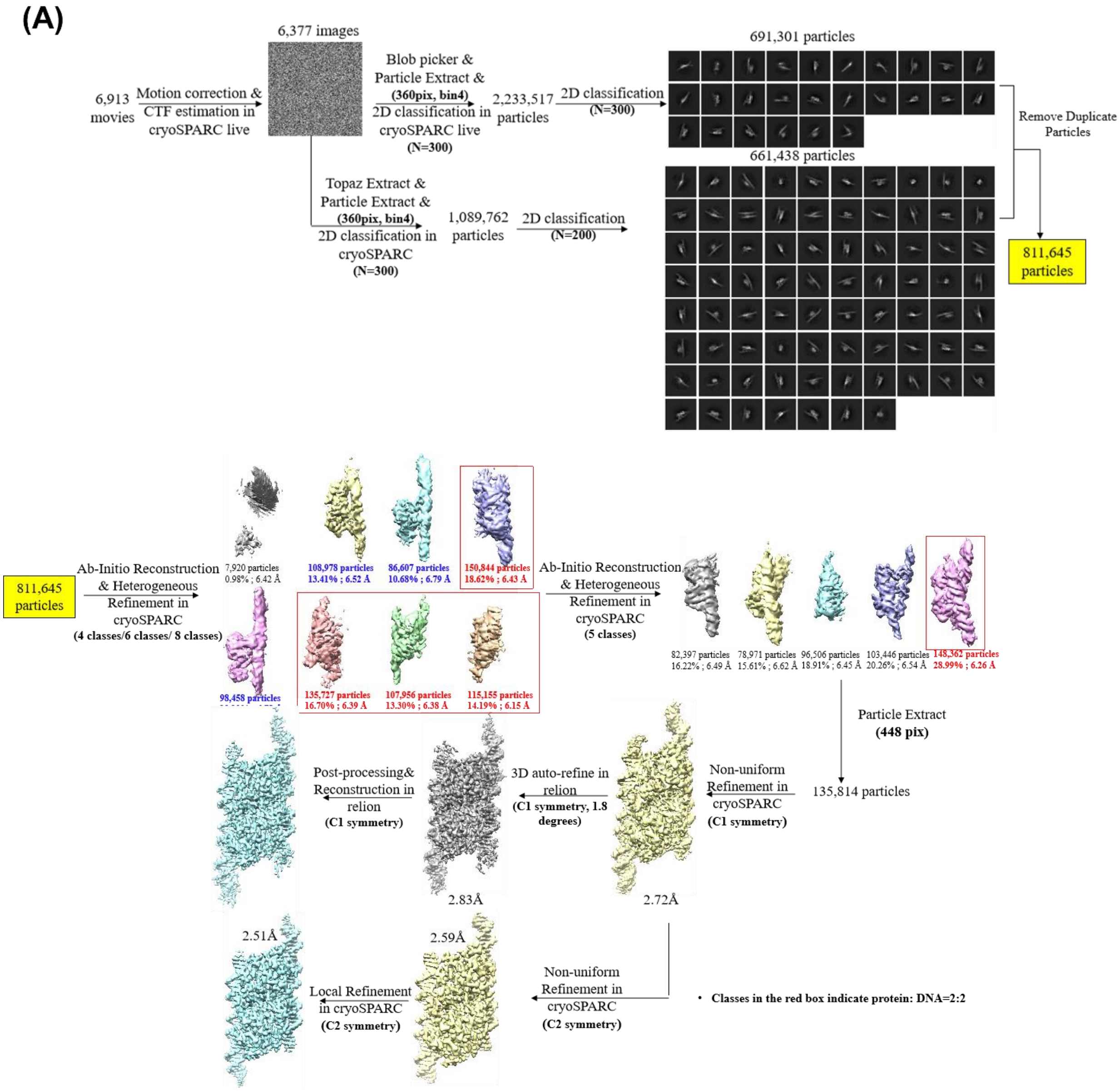

Figure S3: Cryo-EM data processing summary. The flowchart illustrates the key steps involved in processing the Cryo-EM data, spanning from particle picking to classification, to the generation of final maps. A selected subset of the initial reference-free 2D class averages and all the intermediate 3D class averages computed during the processing of this dataset are shown. All 3D class averages, selected for subsequent rounds of processing, are boxed in red, and the percentage of number of particles in each of these is shown. Further attempts to process the discarded classes are omitted from this chart for clarity, as these data did not contribute to the final particle set. (A) 2D classes average 6377 micrographs. (B) After Remove Duplicate Particles job, data processing of dimer binding with duplex probe A (Mce3R dimer:DNA=1:2).

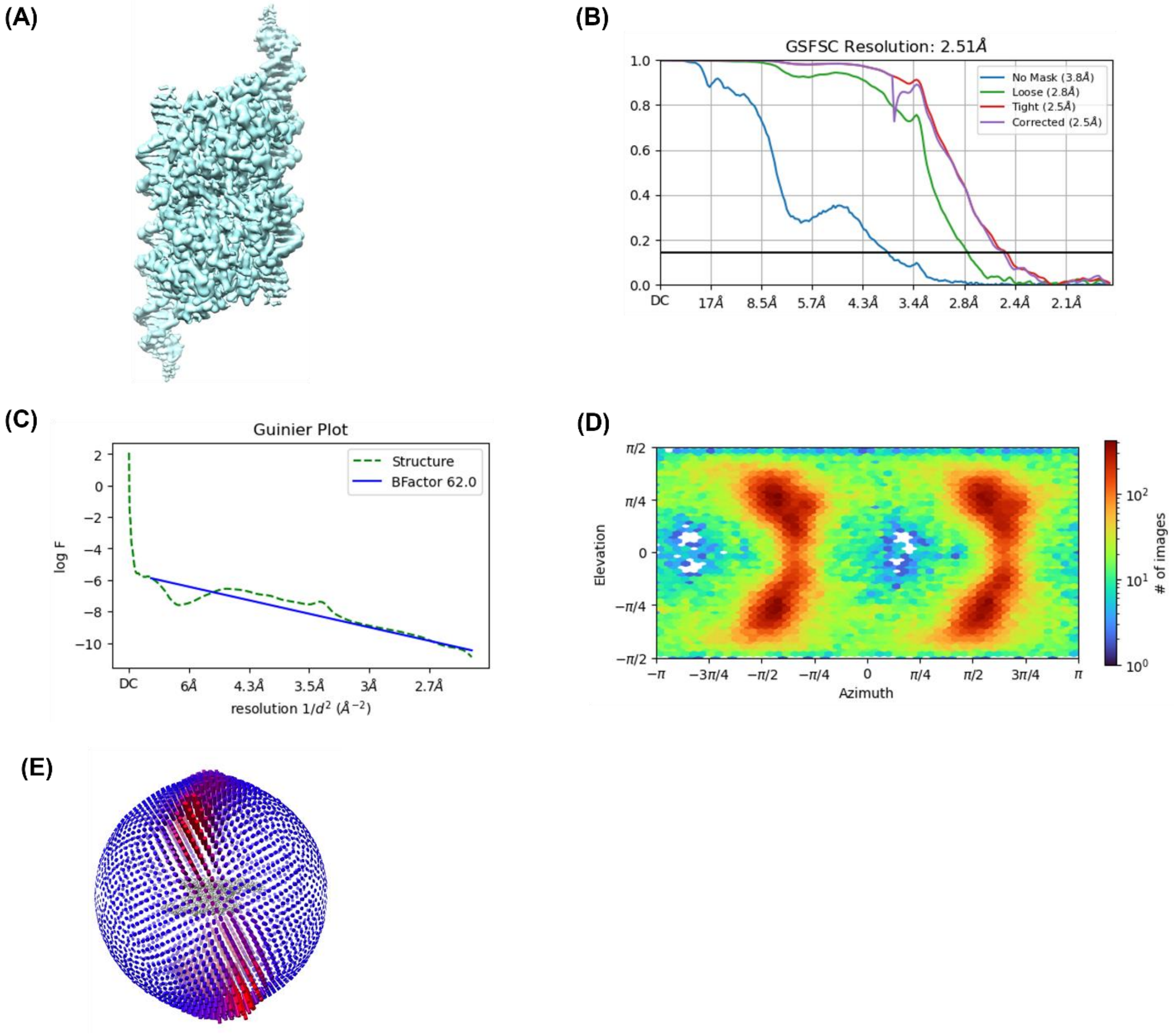

Figure S4: Map and Model quality of Mce3R (Dimer) Binding to Probe A ds-DNA. (A) Cryo-EM density map of the Mce3R/Probe A complex. (B) FSC (Fourier Shell Correlation) Plots representing the final reconstruction's resolution, calculated to be 2.51 Å using the gold standard FSC 0.143 criterion. (C) The B-factor utilized for map sharpening is -62.0 Å², as determined by the Guinier Plot. (D) Viewing Direction Distribution for the final reconstruction. (E) Heat map showing particle orientation distribution.
